# Cryoballoon Ablation of Atrial Fibrillation Through a Permanent Inferior Vena Cava Filter

**DOI:** 10.1002/ccr3.70622

**Published:** 2025-07-10

**Authors:** Ippei Saito, Yuichiro Sagawa, Atsuhito Oda, Hirofumi Arai, Kazuya Murata, Manabu Kurabayashi, Kaoru Okishige, Tetsuo Sasano, Yasuteru Yamauchi

**Affiliations:** ^1^ Department of Cardiology Japan Red Cross Yokohama City Bay Hospital Yokohama Kanagawa Japan; ^2^ Department of Cardiovascular Medicine Institute of Science Tokyo Tokyo Japan

**Keywords:** atrial fibrillation, catheter ablation, cryoballoon, permanent inferior vena cava filter, transfemoral approach

## Abstract

Ablation using a cryoballoon can minimize the number of sheaths passing through a permanent inferior vena cava filter (pIVCF). Cryoballoon ablation with only a few sheaths can be safely and efficiently performed via the usual femoral vein approach in patients with atrial fibrillation and pIVCF.

**Abbreviations**
AF: atrial fibrillation
ICE: intracardiac echocardiography
LA: left atrium
pIVCF: permanent inferior vena cava filter
PVI: pulmonary vein isolation

## 1 Introduction

A permanent inferior vena cava filter (pIVCF) is used to treat pulmonary thromboembolism and deep venous thrombosis when the patient cannot tolerate anticoagulation or when anticoagulant therapy has failed [1]. In the past, pIVCFs were frequently implanted, not only because of their ease of manipulation but also to expand clinical indications [2, 3]. Therefore, patients with atrial fibrillation (AF) commonly have a history of pIVCF implantation. Pulmonary vein isolation (PVI) is a standard treatment for AF [4]; however, it typically requires a transfemoral approach. Although previous studies have shown that radiofrequency catheter ablation of AF through a pIVCF can be performed without complications [5, 6], some operators may hesitate to perform PVI in AF patients with a pIVCF because of potential risks, such as filter erosion, fracture, or dislodgement during wire or sheath manipulation [7]. AF ablation using fewer sheaths and wires introduced through pIVCFs is recommended to prevent pIVCF‐related complications. This paper reports a case of AF that was successfully treated by cryoballoon ablation performed through a pIVCF.

## 2 Case Presentation

### 2.1 Case History

A 52‐year‐old male was referred to our hospital for the treatment of AF. The patient had undergone temporary inferior vena cava filter implantation for pulmonary thromboembolism and deep venous thrombosis 20 years earlier; however, the filter could not be retrieved because of thrombus formation. Consequently, transthoracic thrombectomy was performed, and a pIVCF was implanted. The patient was admitted to our hospital for catheter ablation for AF, which caused palpitations that impaired activities of daily living. The patient had a history of hypertension, dyslipidemia, type 2 diabetes mellitus, hypothyroidism, and obstructive sleep apnea. The patient had been receiving factor Xa inhibitor apixaban at a dose of 10 mg/day prior to the procedure.

### 2.2 Investigations and Treatment

Transthoracic echocardiography revealed a normal left ventricular ejection fraction (62%) and dilated left atrial diameter (47 mm). Computed tomography revealed four pulmonary veins with no anatomical abnormalities, and the left atrial volume was 108 cc with no thrombus. Additionally, the pIVCF was positioned below the renal vein level without thrombus formation.

PVI using a cryoballoon rather than a radiofrequency catheter through the pIVCF was considered to reduce the number of sheaths crossing the pIVCF. The procedure was performed under deep sedation with propofol, and an activated clotting time of 300–400 s was maintained with a continuous infusion of heparin throughout the procedure. The perioperative anticoagulation regimen was implemented with slight modifications to the protocol previously reported by our institution [8]. Specifically, apixaban was switched to dabigatran on the evening of the admission day, and dabigatran was continued throughout the hospitalization period. On the evening following discharge, the anticoagulant was switched back to the original regimen of apixaban.

**FIGURE 1:**
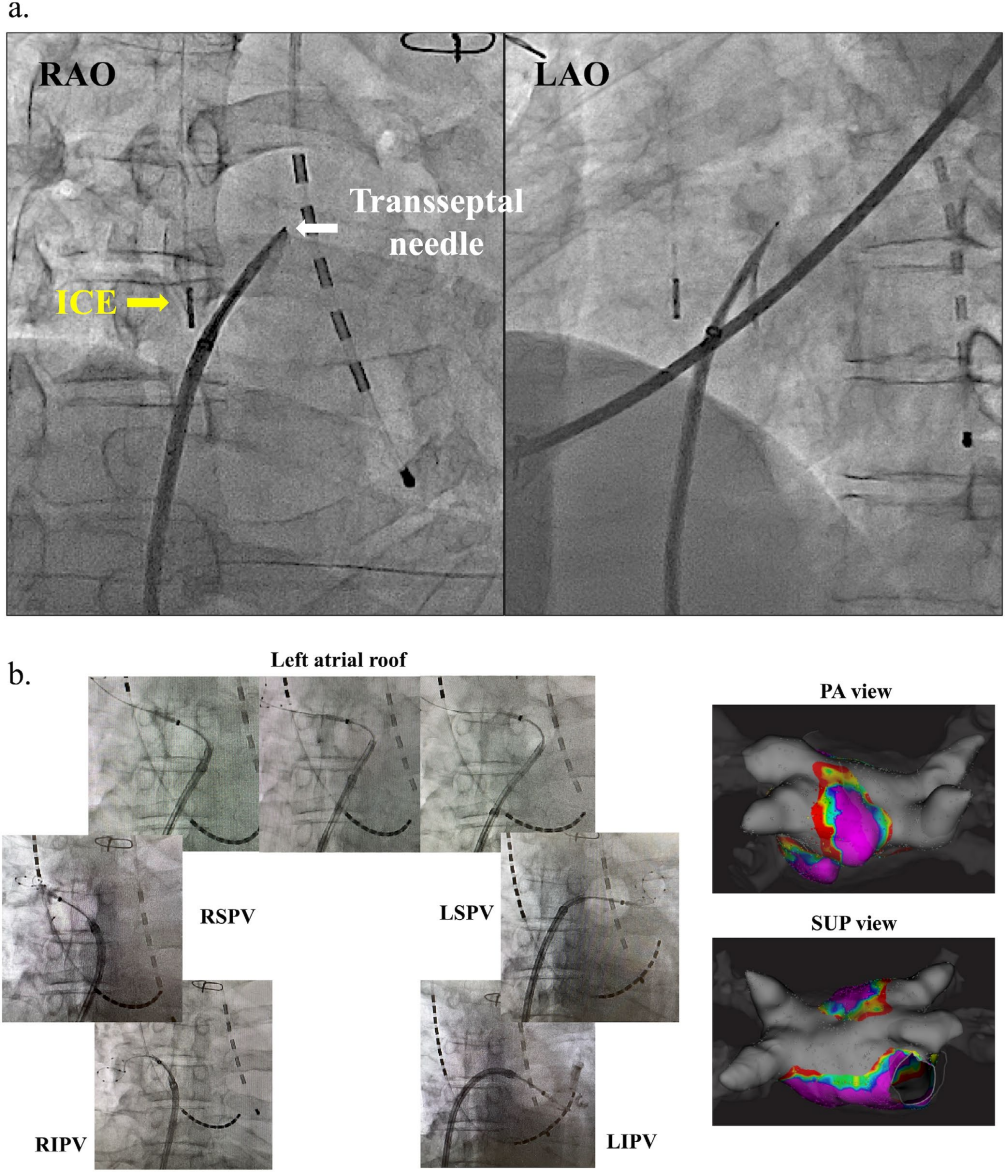
Atrial septal puncture and ablation procedure. (a) An atrial septal puncture was performed under the guidance of an intracardiac echocardiography (ICE) catheter, which was inserted through the right jugular vein. (b) PVI and left atrial roof‐line ablation were successfully carried out using a cryoballoon. Post‐ablation images recorded with electroanatomical mapping are shown. The voltage map indicates the completion of PVI and scarring of the left atrial roof. The purple regions represent a voltage greater than 0.5 mV, while the gray regions indicate a voltage less than 0.1 mV. ICE, intracardiac echocardiography; LAO, left anterior oblique; LIPV, left inferior pulmonary vein; LSPV, left superior pulmonary vein; PA, posterior–anterior; PVI, pulmonary vein isolation; RAO, right anterior oblique; RIPV, right inferior pulmonary vein; RSPV, right superior pulmonary vein; SUP, superior.

An 8‐Fr sheath was placed in the right internal jugular vein, and an intracardiac echocardiography (ICE; AcuNav: Biosense Webster) catheter was inserted through this sheath. The right femoral vein was punctured, and a 0.032‐in. J‐tip guidewire was carefully passed through the pIVCF (Video S1). An 8.5‐Fr sheath (SL‐0) was inserted through the pIVCF via the right femoral vein (Video S2). An atrial‐septal puncture was performed using a transseptal needle (NRG transseptal needle; Baylis Medical) under the guidance of an ICE catheter, which was inserted through the right internal jugular vein to minimize the risk of complications associated with the presence of a pIVCF (Figure 1a). Following the puncture, an IntellaMap Orion 64 mini‐electrode basket catheter (Boston Scientific) was inserted into the left atrium (LA) via the right femoral vein. After ICE catheter removal, a coronary sinus‐high right atrium‐superior vena cava electrode catheter (BeeAT; Japan Lifeline) was inserted into the coronary sinus via the right internal jugular vein. Electroanatomical mapping was performed to confirm the shape and voltage of the entire LA during atrial burst pacing. The transseptal sheath was exchanged over the guidewire for a 15.9‐Fr steerable sheath (POLARSHEATH, Boston Scientific). The 15.9‐Fr sheath was successfully passed through the pIVCF without complications (Video S3). Sheath advancement was performed slowly over a stable wire under continuous fluoroscopic guidance to avoid contact with or deformation of the filter. Manual resistance and catheter feedback were closely monitored throughout the procedure. PVI and left atrial roof line ablation were completed using a cryoballoon catheter (POLARx; Boston Scientific), as previously described (Figure 1b) [9]. No additional radiofrequency touch‐up ablations were performed. Notably, the position of the pIVCF remained stable after the removal of the 15.9‐Fr sheath (Video S4). Finally, a contrast medium was injected through the 15.9‐Fr sheath, confirming proper positioning of the pIVCF without any deformation, dislodgement, or bleeding from the inferior vena cava.

### 2.3 Outcome and Follow‐Up

Following ablation, the patient was followed up without the use of antiarrhythmic drugs. Sinus rhythm was maintained for approximately 2 years of follow‐up, and the patient was doing well.

## 3 Discussion

Although the Superior vena cava approaches accessing the LA without interfering with the pIVCF, in which the internal jugular or subclavian vein is punctured, have been used as alternative routes [10, 11], they may be associated with several limitations. These factors include increased procedural complexity, reduced catheter maneuverability, and a steeper learning curve, particularly for operators who are less experienced with non‐femoral access. In contrast, the conventional femoral approach provides more familiar catheter trajectories, better control during ablation, and alignment that is more compatible with standard cryoballoon workflows. Given these differences, a conventional approach through the right femoral vein was adopted in this case.

In a report of 40 patients who underwent catheter ablation through a pIVCF, radiofrequency ablation was performed in 21 patients with AF [7]. Long sheaths such as Agilis, SL‐0, SL‐1, and LAMP90 were used in 35 cases, with a mean of 1.63 ± 0.49 sheaths per case. No complications were associated with the ablation procedure. This report indicates that the radiofrequency energy system may be regarded as a safe therapeutic option for the ablation of AF complicated by the presence of pIVCF. However, we chose the cryoballoon ablation system over radiofrequency ablation to minimize the number of sheaths and catheter manipulations across the pIVCF. While radiofrequency ablation typically requires multiple catheters, such as an ablation catheter and a circular mapping catheter, cryoballoon ablation enables pulmonary vein isolation using a single balloon catheter, thereby simplifying the procedure. Consequently, cryoballoon ablation may be considered a viable treatment option.

**FIGURE 2:**
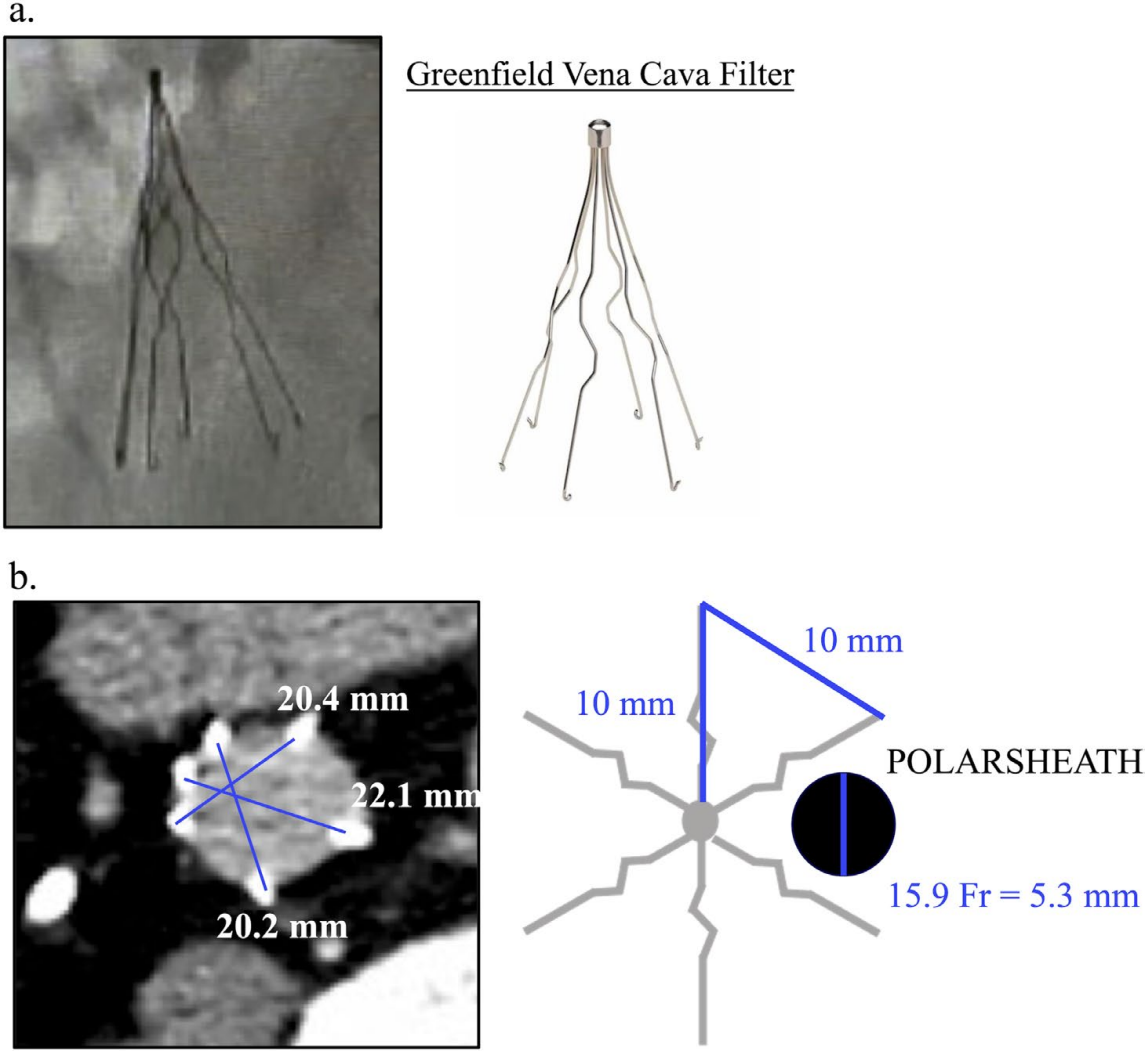
Sizes of pIVCF and POLARSHEATH. (a) We assumed that the pIVCF in this patient was a Greenfield–Vena Cava filter. (b) The radius of the pIVCF on the computed tomography image was approximately 20 mm. Because the diameter of the POLARSHEATH was approximately 5.3 mm, there seemed to be sufficient space around the sheath. pIVCF, permanent inferior vena cava filter.

To the best of our knowledge, this is the first case report of successful cryoballoon ablation for AF, in which a large‐caliber sheath was required for a patient with an implanted pIVCF. A cryoballoon catheter was introduced through the right femoral vein, and an ICE catheter was inserted through the right internal jugular vein, reducing the number of sheaths crossing the pIVCF. A 0.032‐in. J‐tip guidewire packaged with an SL0 sheath was used to traverse the pIVCF, as it features an atraumatic tip and is commonly employed for venous navigation. In this case, the filter's structure allowed for smooth passage without resistance; therefore, a specialized wire, such as an angled or hydrophilic wire, was not necessary. However, in cases involving more complex filter configurations, alternative wires may offer improved trackability and safety. Although the POLARSHEATH is a large‐caliber sheath (15.9‐Fr), we were able to manipulate the wires and sheaths with minimal interference with the pIVCF due to the sufficient space between the sheath and the pIVCF (Figure 2). Specific spaces between the pIVCF struts were identified using fluoroscopy in the left anterior oblique (LAO) view during the procedure, and the wire was navigated through the most favorable trajectory. However, in anatomically complex filters, such as the one observed in this case, relying solely on the LAO view may be insufficient. Additional confirmation using the right anterior oblique (RAO) or left lateral view could have been beneficial in ensuring optimal visualization and navigation through these spaces.

Potential complications in similar clinical scenarios may include filter displacement, thrombus dislodgement, or mechanical damage to the filter or surrounding vasculature. These risks underscore the importance of careful preprocedural imaging, meticulous sheath handling, and real‐time fluoroscopic monitoring.

Although this case demonstrates the feasibility of cryoballoon ablation in a patient with pIVCF, it is important to note that this is a single‐case report, which inherently limits its generalizability. Additionally, procedural complexity and risk may vary depending on the type, size, and design of the implanted IVC filter, warranting further investigation. The cryoballoon approach offers advantages such as procedural simplicity and shorter procedure times, which may be beneficial for patients with pIVCF. However, its reliance on a relatively large‐caliber sheath may present mechanical challenges in cases with limited intravascular space or complex filter anatomy. While the two‐year follow‐up in this case was uneventful, we acknowledge that further studies are needed to validate the long‐term safety and efficacy of cryoballoon ablation in patients with implanted pIVCFs.

In conclusion, cryoballoon ablation via the conventional femoral approach may be feasible and beneficial for AF patients with pIVCFs without increasing the risk of complications.

## Author Contributions

**Ippei Saito:** writing – original draft, writing – review and editing. **Yuichiro Sagawa:** methodology, supervision, writing – original draft, writing – review and editing. **Atsuhito Oda:** writing – review and editing. **Hirofumi Arai:** writing – review and editing. **Kazuya Murata:** writing – review and editing. **Manabu Kurabayashi:** writing – review and editing. **Kaoru Okishige:** writing – review and editing. **Tetsuo Sasano:** writing – review and editing. **Yasuteru Yamauchi:** conceptualization, supervision, writing – review and editing.

## Consent

Written informed consent was obtained from the patient to publish this report in accordance with the journal's patient consent policy.

## Conflicts of Interest

The authors declare no conflicts of interest.

## Supporting information


**Video S1.** Guidewire carefully crossing the pIVCF via the right femoral vein.

**Video S2.** Long sheath inserted through the pIVCF.

**Video S3.** Successful passage of a 15.9‐Fr cryoballoon sheath through the pIVCF without complications.

**Video S4.** Removal of the 15.9‐Fr sheath with no change in pIVCF location.

## Data Availability

The data underlying this article will be shared upon reasonable request to the corresponding author.
