# Supplementary figures and images for: Cryoballoon Ablation of Atrial Fibrillation Through a Permanent Inferior Vena Cava Filter
Source: Clin Case Rep. 2025 Jul 10;13(7):e70622. doi: 10.1002/ccr3.70622 (PMC12245721; doi:10.1002/ccr3.70622)

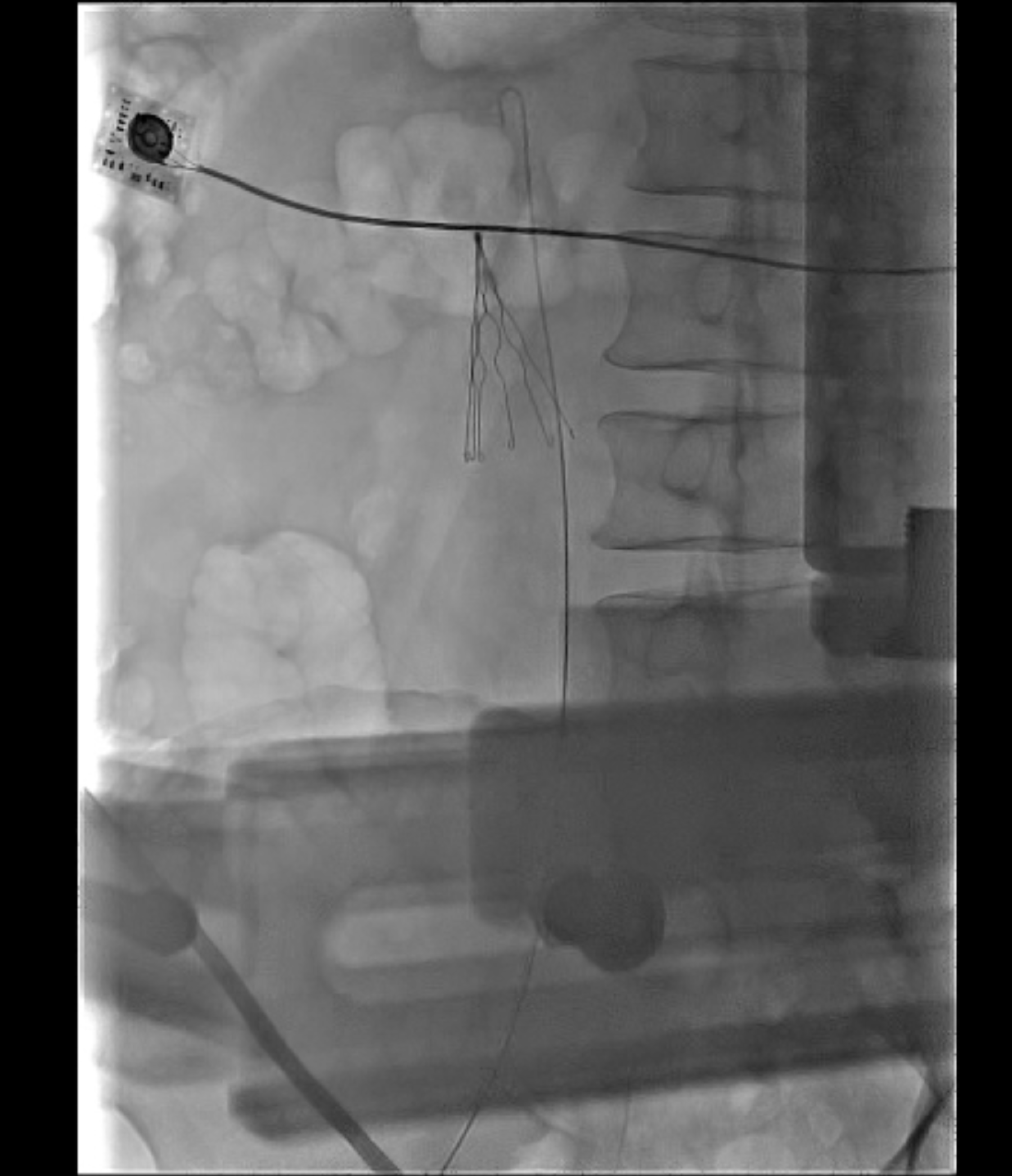

Supplement: Supplementary file 1 — Video S1. Guidewire carefully crossing the pIVCF via the right femoral vein. [file CCR3-13-e70622-s003.zip › CCR3_70622_f1_Video1_Place-holder.png]

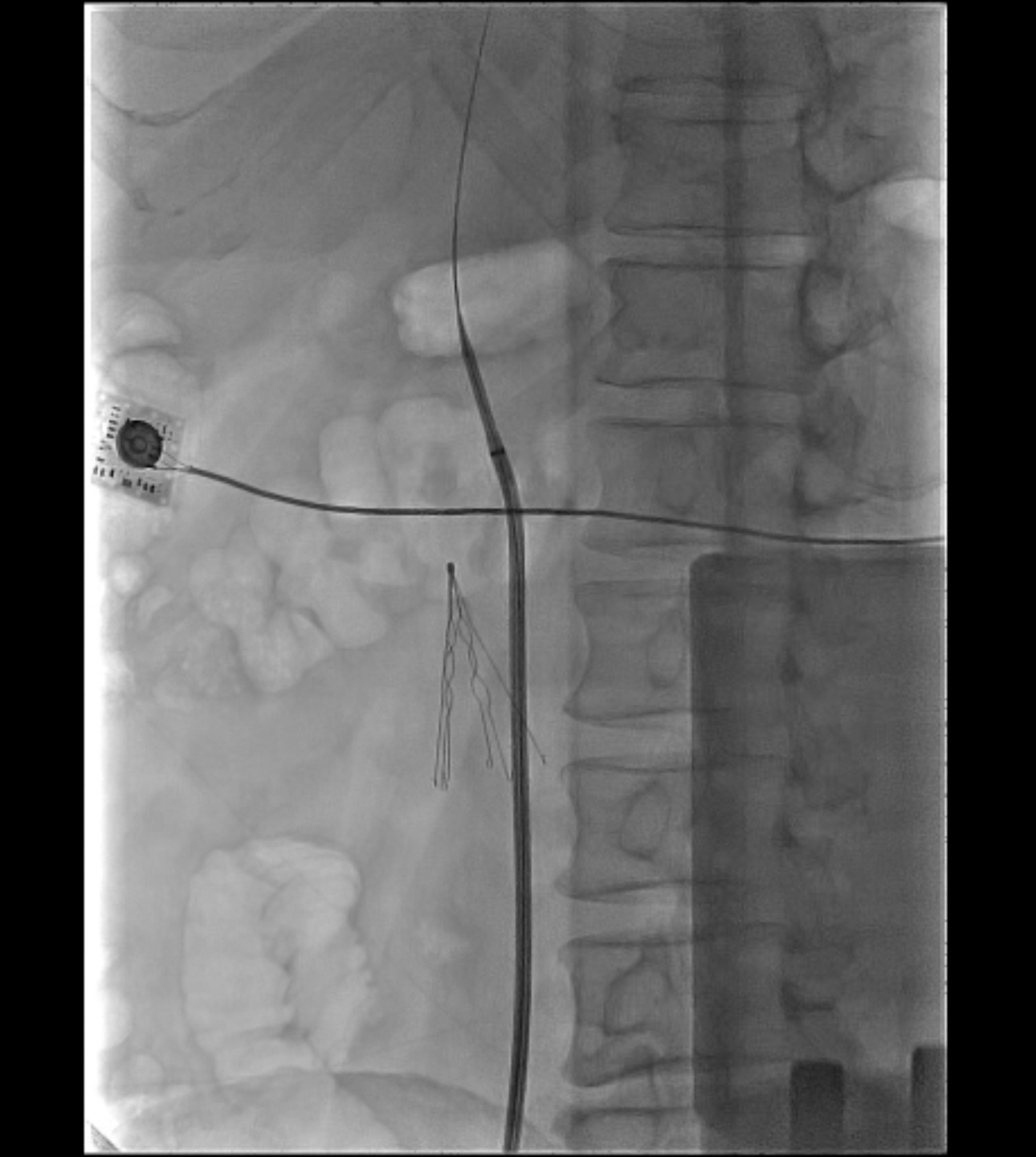

Supplement: Supplementary file 2 — Video S2. Long sheath inserted through the pIVCF. [file CCR3-13-e70622-s004.zip › CCR3_70622_f2_Video2_Place-holder.png]

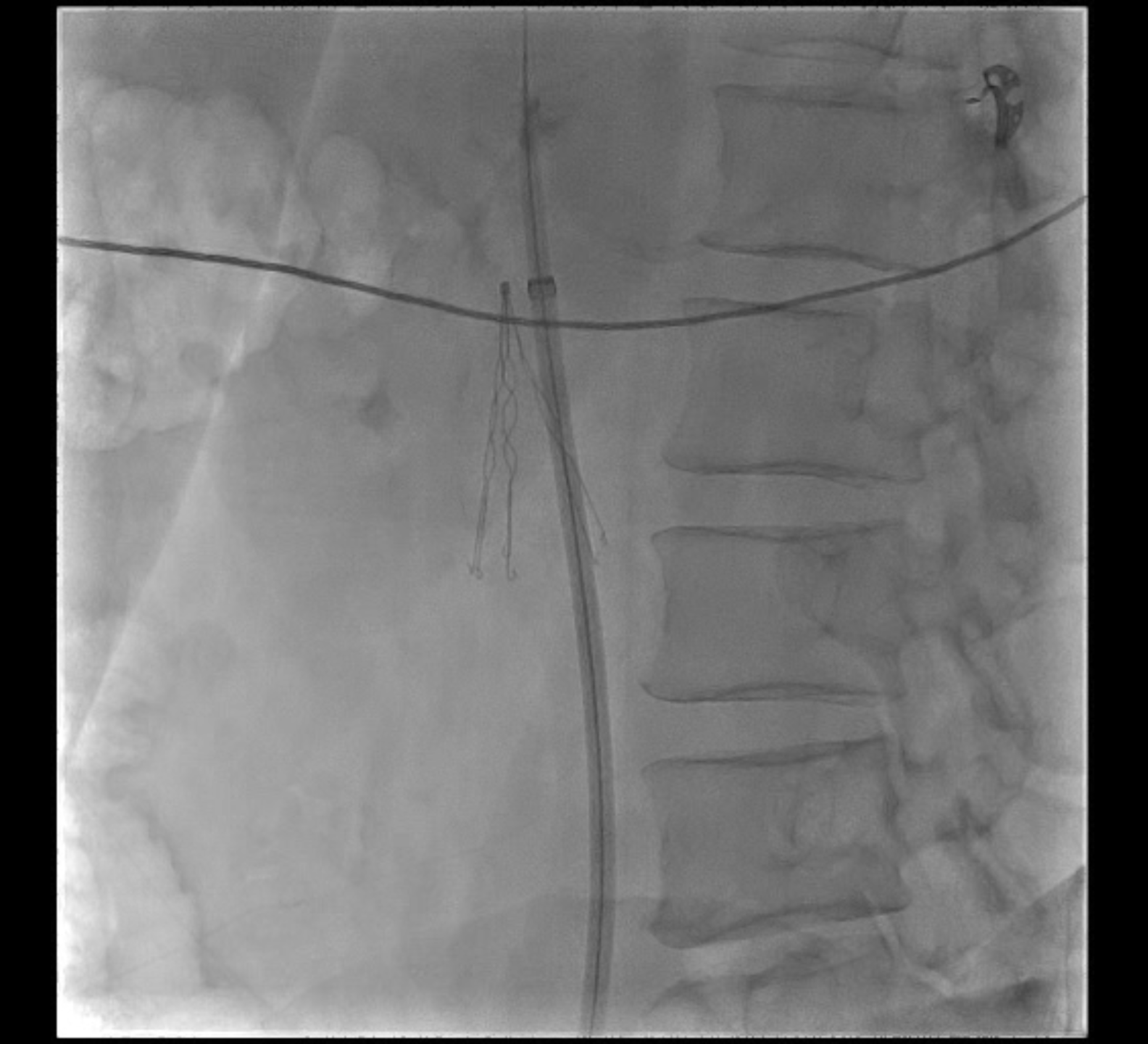

Supplement: Supplementary file 3 — Video S3. Successful passage of a 15.9‐Fr cryoballoon sheath through the pIVCF without complications. [file CCR3-13-e70622-s002.zip › CCR3_70622_f3_Video3_Place-holder.png]

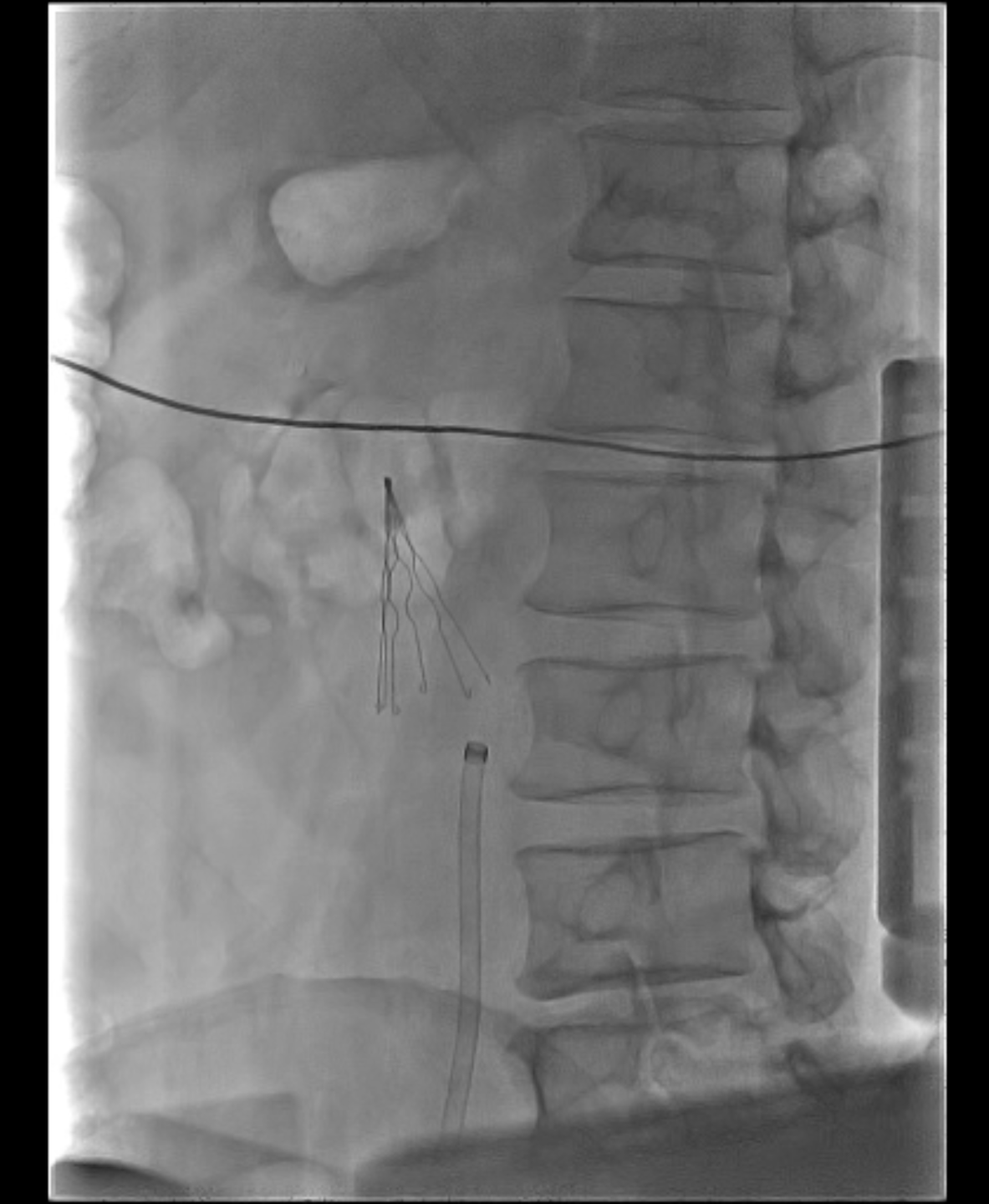

Supplement: Supplementary file 4 — Video S4. Removal of the 15.9‐Fr sheath with no change in pIVCF location. [file CCR3-13-e70622-s001.zip › CCR3_70622_f4_Video4_Place-holder.png]
